# Enhancing Doctors’ Competencies in Communication With and Activation of Older Patients: The Promoting Active Aging (PRACTA) Computer-Based Intervention Study

**DOI:** 10.2196/jmir.6948

**Published:** 2017-02-22

**Authors:** Dorota Wlodarczyk, Joanna Chylińska, Magdalena Lazarewicz, Marta Rzadkiewicz, Mariusz Jaworski, Miroslawa Adamus, Gørill Haugan, Monica Lillefjell, Geir Arild Espnes

**Affiliations:** ^1^ Second Faculty of Medicine Department of Medical Psychology Medical University of Warsaw Warsaw Poland; ^2^ NTNU Center for Health Promotion Research Department of Public Health and Nursing Norwegian University of Science and Technology Trondheim Norway; ^3^ NTNU Center for Health Promotion Research Department of Neuromedicine and Movement Science Norwegian University of Science and Technology Trondheim Norway

**Keywords:** health services for the aged, active aging, e-learning, general practioners, professional competence, psychosocial competencies, health communication, seniors’ expectations, seniors’ attitude toward treatment and health

## Abstract

**Background:**

Demographic changes over the past decades call for the promotion of health and disease prevention for older patients, as well as strategies to enhance their independence, productivity, and quality of life.

**Objective:**

Our objective was to examine the effects of a computer-based educational intervention designed for general practitioners (GPs) to promote active aging.

**Methods:**

The Promoting Active Aging (PRACTA) study consisted of a baseline questionnaire, implementation of an intervention, and a follow-up questionnaire that was administered 1 month after the intervention. A total of 151 primary care facilities (response rate 151/767, 19.7%) and 503 GPs (response rate 503/996, 50.5%) agreed to participate in the baseline assessment. At the follow-up, 393 GPs filled in the questionnaires (response rate, 393/503, 78.1%), but not all of them took part in the intervention. The final study group of 225 GPs participated in 3 study conditions: e-learning (knowledge plus skills modelling, n=42), a pdf article (knowledge only, n=89), and control (no intervention, n=94). We measured the outcome as scores on the Patients Expectations Scale, Communication Scale, Attitude Toward Treatment and Health Scale, and Self-Efficacy Scale.

**Results:**

GPs participating in e-learning demonstrated a significant rise in their perception of older patients’ expectations for disease explanation (Wald χ^2^=19.7, *P*<.001) and in perception of motivational aspect of older patients’ attitude toward treatment and health (Wald χ^2^=8.9, *P*=.03) in comparison with both the control and pdf article groups. We observed additional between-group differences at the level of statistical trend. GPs participating in the pdf article intervention demonstrated a decline in self-assessed communication, both at the level of global scoring (Wald χ^2^=34.5, *P*<.001) and at the level of 20 of 26 specific behaviors (all *P*<.05). Factors moderating the effects of the intervention were the number of patients per GP and the facility’s organizational structure.

**Conclusions:**

Both methods were suitable, but in different areas and under different conditions. The key benefit of the pdf article intervention was raising doctors’ reflection on limitations in their communication skills, whereas e-learning was more effective in changing their perception of older patients’ proactive attitude, especially among GPs working in privately owned facilities and having a greater number of assigned patients. Although we did not achieve all expected effects of the PRACTA intervention, both its forms seem promising in terms of enhancing the competencies of doctors in communication with and activation of older patients.

## Introduction

### Why Is Activation of Older Patients Important While Practicing Medicine?

Demographic changes over the past decades have created new challenges for health care providers [1]. Current trends in aging, along with continued limitations in providing effective health care to older patients, make it necessary to offer activities that promote health and disease prevention for older people, as well as strategies to enhance their independence, productivity, and quality of life [2,3].

Older patients’ use of health care services is increasing [3], but certain aging processes may be modified by individual activity. Thus, a general practitioner’s (GP) office appears to be the right place to discuss desired health- and activity-enhancing behaviors. Patients have been shown to depend on clinicians in areas such as preserving and promoting physical and emotional health [4]. Individuals who had been helped by a health provider to learn how to monitor their condition and to set goals were more activated than those who didn’t receive such assistance [5,6]. At the same time, preventive health behavior and related matters have rarely been addressed by physicians and patients over 60 years of age in the primary health care setting [7].

Moreover, the lack of supporting patient activation and engagement was determined to be a potential pitfall for institutions aiming at improving quality and reducing health care costs [8].

### What Might Activation of Older Patients by GPs Involve?

Patient activation is defined as the patient’s ability to self-manage symptoms and problems, involvement in clinical decision making, and engagement in activities that maintain functioning and reduce health decline [9]. Its measurement allows their doctors to assign patients to 1 of 4 levels of activation, ranging from passive to proactive [10,11].

The unidimensional approach, however, does not allow for estimating the level of certain components of attitude toward treatment and health (ATH), such as cognitive, emotional (including positive and negative aspects), and motivational components [12,13]. An increasing number of studies have also shown the significance of self-efficacy in initiating and maintaining health behaviors [14].

To successfully activate older patients, it is necessary to identify their expectations of both the medical encounter [15] and effective communication [16,17]. Initial attempts to categorize patients’ needs demonstrated the importance of explaining disease states, providing emotional support, and acting on or providing information about medical treatment [18]. As far as successful and active aging is concerned, the need for information about health promotion opportunities and improvement of quality of life is growing in importance [19].

In turn, accurate recognition of older patients’ beliefs about their GPs’ communication is one of the most important factors contributing to good physician-patient rapport [20,21]. Patient-centered communication, taking into account older people’s perspective and empowering them in the process of decision making, was found to be associated with more favorable outcomes for patients [22,23]. Communicational aspects of the encounter are frequently rated by patients as the most important, although specificity of expectations depending on the medical situation has been found [24].

People over 65 years of age are seen through the prism of age-related stereotypes picturing them as poor, frail, and dependent [25-27]. Furthermore, there is an increasing number of studies on GPs’ beliefs concerning older patients [28,29]. Such studies have taught us that physicians’ attitudes toward elderly patients are diverse and positively related to factors such as geriatrics training, which suggests that providing GPs with appropriate knowledge and skills is essential [30]. Ageist misconceptions of older patients as indifferent, rigid in their beliefs and practices, or frequently annoyed may have serious negative consequences for them [31].

### What Do We Know About E-Learning Versus Traditional Learning of GPs?

Selecting a teaching approach that both is relevant for the group and serves educational objectives is of significant importance, especially in the case of doctors, who are frequently overworked, lack time, and face changing demands. E-learning can be an attractive alternative to traditional learning. It is defined as any educational intervention that is mediated electronically via the Internet asynchronously [32].

The benefits of e-learning have been reported in terms of increased accessibility to education, improved self-efficacy, knowledge generation, cost effectiveness, learner flexibility, and interactivity; reports regarding its usage and effectiveness, however, have ranged broadly [33]. E-learning has proved to be at least as effective as traditional learning in terms of knowledge acquisition and user satisfaction, although knowledge about its effects on behavior change and patient outcomes is still insufficient [32]. A systematic review led to the conclusion that situated e-learning (embedded within a specific context representing real practice) enhances novice learners’ performance, but that its effect on knowledge improvement is limited when compared with traditional learning [34]. There is also little knowledge about e-learning effectiveness in teaching GPs psychosocial competencies, such as communication and older patient activation. Moreover, little has been done to improve the knowledge about factors that moderate outcomes of different types of interventions [33].

### Research Problems

Considering the above, we developed and investigated the Promoting Active Aging (PRACTA) intervention for GPs. It was designed to stimulate better recognition of older patients’ expectations related to the medical encounter, more effective and patient-centered communication with older patients, and a better ability to enhance active ATH among older patients. We rooted the content of the intervention in selected theories in the field of health psychology, such as successful aging theories [35,36], attitude toward health [12], patient-centered communication [37], models of health behavior change [38], or social support theories [39], as well as the results of surveys from doctors and patients collected at time 1 of this study.

We presented the intervention in 2 forms: an e-learning platform and an article in pdf format. The e-learning intervention was a multimedia program aimed at presenting knowledge and modelling skills (demonstration of recommended solutions by means of, for example, case studies and video-recorded dialogues, and testing of new skills in simulated situations). The pdf article was a digitized text with pictures aimed solely at presenting knowledge. First, we anticipated that gaining some knowledge about older patients’ expectations and ways of enhancing their active attitude toward health might contribute to a more positive perception of older patient activation among GPs, whereas practicing skills might cause more changes in GPs’ behavior, thus initiating a positive spiral of changes (GPs use the skills of older patient activation, leading to older patients becoming more active, as a result of which GPs appraise older adults as being capable of being active, and consequently GPs continue older patient activation). Second, we assumed that, due to adoption of a wider range of didactic tools, e-learning would have a stronger and broader effect (in terms of number of outcome variables) than the pdf article.

We enrolled doctors in 1 of 3 study groups: e-learning, pdf article (comparative group), and control (no intervention at this time). The aim of the study was to examine the short-term effects of the PRACTA educational intervention in reference to the following outcome variables: (1) GPs’ perception of older patients’ expectations of the medical encounter, (2) GPs’ self-assessed communication skills, and (3) GPs’ perception of older patients’ ATH.

We formulated 2 research questions (RQs), as follows. RQ1: Do the study groups differ from each other in terms of changes observed in the outcome variables? We hypothesized that, among the GPs in the intervention groups (e-learning and pdf article), in comparison with the control group, there would be (1a) a greater increase in their perception of older patients’ expectations related to different types of information, with the increase in the e-learning group being larger than in the pdf article group (we had no directional hypotheses about other types of expectations), (1b) a greater increase in self-assessed communication skills, with the increase in the e-learning group being larger than in the pdf article group, (1c) a greater increase in their perception of older patients’ active ATH, with the increase in the e-learning group being larger than in the pdf article group. RQ2: Are there any factors affecting the effects of the PRACTA intervention? Due to the exploratory nature of the question, we did not formulate any directional hypotheses but assumed that some sociodemographic, vocational, and organizational factors that we took into account would be moderators of the effects of the intervention.

## Methods

### Procedure and Recruitment

The PRACTA study consisted of a baseline questionnaire administered to GPs (time 1), implementation of the intervention (available for 3 months), and follow-up questionnaire administered to the GPs (time 2, which took place 1 month after the intervention). Recruitment comprised 2 stages: recruitment of primary care facilities (we contacted the facility’s management to obtain permission to recruit the GPs) and recruitment of GPs working in these facilities. We considered the following inclusion criteria for facilities: delivering primary care, having a contract with the National Health Fund (patients did not pay for services out of their private funds), and being located in central Poland (a slightly wealthier part of the country including both urban and rural areas). The inclusion criteria for doctors were as follows: working in a facility recruited for the study, delivering primary care, and signing a written consent to participate in all parts of the project. The procedure guaranteed that data collection would be depersonalized, and every single GP was instructed in how to create an individual code that enabled matching scores from time 1 and time 2.

**Figure 1:**
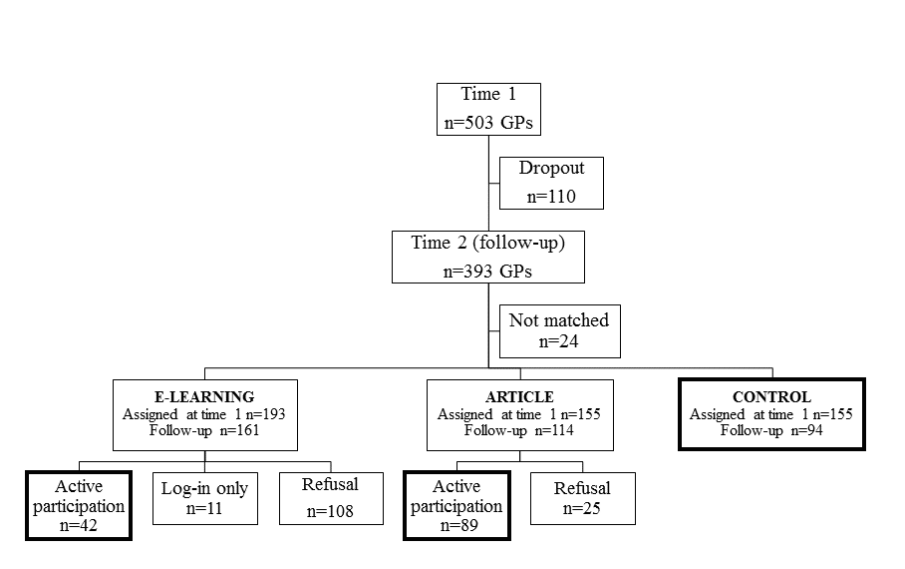
Flow chart of participation in the Promoting Active Aging (PRACTA) intervention study. GP: general practitioner.

A total of 151 primary care facilities (response rate 151/767, 19.7%) and 503 GPs (response rate 503/996, 50.5%) agreed to participate in the baseline assessment. The facilities were randomly assigned to 3 groups: e-learning, pdf article, and control (we used random assignment of facilities only to ensure that all GPs working in the facility had access exclusively to one type of intervention). At time 2, there was a 78.1% response rate (393/503), but in 24 cases a missing or wrong individual code made matching scores from both measurements impossible (we considered these cases as dropouts). Figure 1 presents a flowchart of GP participation in consecutive stages of the project.

The final study sample consisted of 225 GPs: 42 actively taking part in e-learning (logged in and received points in at least one test), 89 actively participating in the pdf article intervention (filling out the form with questions regarding the pdf article that was an indicator of active participation), and 94 constituting the control group (participating in time 1 and time 2 surveys without any intervention at this time). As Figure 1 shows, 11 (21%) of 53 GPs logged in only, without receiving any points, but of the remaining 42 who obtained some points, 30 (71%) had completed all 5 modules, 2 (5%) had completed 4 modules, and 10 (24%) had completed 3 or fewer modules. The pdf article group was asked about what part of the article they had read: 47 (53%) declared that they had read the article in full, 19 (21%) had read three-quarters of the text, 10 (11%) had read approximately half of the article, and 13 (15%) had read approximately one-quarter or less. Whenever possible, interviewers asked doctors why they had declined to participate in the intervention, and “lack of time” was the most frequent response.

All GPs from the pdf article and control groups who declared their willingness to participate in PRACTA e-learning got access to it after completing the time 2 assessment. Doctors were interviewed by professional interviewers who had been trained on project-specific requirements for standardization of assessments. The content and form of both types of intervention were developed by researchers who prepared the whole project and carried out time 1 analyses. Approval for the study was obtained from the Bioethics Committee of the Medical University of Warsaw.

### PRACTA Intervention

We developed the PRACTA intervention in 2 forms. First, we prepared the e-learning intervention and then, based on its content, we created a pdf article. Both forms included 5 modules that were identically themed and presented in the same order; they were different, however, in their range, volume, and teaching approaches to present both knowledge and skills.

The modules covered the following subjects: (1) the process of active aging and the importance of an active attitude toward health, (2) doctors’ beliefs about older adults’ abilities and expectations, (3) the importance of physician-patient rapport for older patients and their health outcomes, (4) psychological rules and skills for promoting an active attitude toward health, and (5) quality of life and providing support for older patients (see Multimedia Appendix 1 for a detailed description of both types of intervention).

E-learning was designed to be a game in which participating players chose their character (female or male) and then receive specific task missions to complete. It included various multimedia components, which allowed for demonstration of specific practical solutions and for modelling communication and older adults’ activation skills. One module took about 1 hour to compete. To join the e-learning intervention, each participant was given a personal log-in and password and a USB flash drive with the information about time to access and rules of conduct.

The pdf article intervention took the form of a text with concise information, divided into small sections, and structured visually with simple pictures and figures (all images used in the pdf version were extracted from the e-learning intervention). Information presented in the pdf article had the form of a summary of the e-learning content and included a general description of solutions and techniques. Each pdf article module had a length of 3 pages of A4-sized paper. To join this form of intervention, each participant was given a USB flash drive with the article in pdf format.

Before the intervention started, we had launched an information campaign in primary care facilities (through letters sent to facility heads and their representatives who would be responsible for the PRACTA project-facility collaboration) and among GPs. The campaign for GPs comprised 2 phases: in phase 1, we mailed letters to GPs informing them about the timetable of scheduled stages of the project; in phase 2, interviewers visited GPs to deliver verbal information, leaflets, and USB flash drives with instructions and personal log-ins and passwords. After 4 weeks of the intervention (and evaluation of the response rate), we took other steps to remind and encourage GPs to participate: a letter, a visit by an interviewer, 2-3 telephone calls to a facility representative (until GPs clearly refused to participate) in the e-learning group and a letter only in the pdf article group.

The PRACTA e-learning intervention was administrated via the Medical University of Warsaw website. The e-learning platform (Microsoft SharePoint with SharePoint Learning Kit, version SLK 1.8; Microsoft Corporation) consisted of 2 systems: a learning content management system and a learning management system. To create the e-learning intervention, we used Articulate Storyline software (version 1; Articulate Global, Inc). To access PRACTA e-learning, each GP was given personal log-in and password (with information on time to access, rules of conduct, and availability of technical support). This procedure was very simple and user friendly (there were no requests for assistance). The first page after logging in contained overall information on the structure and format of the entire program. Participants were instructed to begin with module 1 and continue sequentially to finish with module 5. It was possible to stop and resume an uncompleted module at any time (the module was not available to a user only after completing the final test and saving scores). The participants were guided through the PRACTA e-learning by active arrows and written or audio instructions (eg, choose your character, click the button or read the materials, watch a video) with no interactions with a tutor or other participants (unidirectional). Navigating e-learning was easy and intuitive, allowing the users not to concentrate on technical aspects. The system allowed for monitoring the GPs’ log-ins and test results evaluating their knowledge after completion of each module (only after saving the results).

The total number of points was calculated and converted into credit points (registered by the Polish Chamber of Physicians, *Izba Lekarska*, as a form of professional development). A diploma confirming the number of credit points was a major reward for participating in e-learning (certificates of participation in the PRACTA project and small project gadgets were given to all participants).

### Measurement

The outcome variables in the study were (1) the GPs’ perception of older patients’ appointment-related expectations, (2) GPs’ self-assessed communication behavior, and (3) the GPs’ perception of older patients’ ATH. The tools were designed for the purpose of the PRACTA project, and their psychometric properties were evaluated after the pilot study had been completed, and later, at the time of both questionnaires (time 1 and time 2) of the study proper. The questionnaires were originally written and administered in Polish, but their English translations are presented here in the multimedia appendixes.

#### GP’s Perception of Older Patients’ Appointment-Related Expectations

We measured this outcome variable with the PRACTA Patients Expectations Scale-Doctors (PRACTA-PES-D) consisting of six 3-item sections (each section was a single-factor scale) (Multimedia Appendix 2 shows the full scale in English translation). Each section reflected a different expectation: disease explanation, treatment explanation, health advice, quality-of-life improvement, rapport, and emotional support. Example items from each section are as follows: “Usually, during a visit elderly patients (65+) expect me…to find the cause of their symptoms” (disease explanation), “to present the results of the tests performed” (treatment explanation), “to encourage them to make health promoting changes” (health advice), “to suggest ways of maintaining life satisfaction” (quality-of-life improvement),” to show them respect” (rapport), and “to talk to them about how they feel and how they cope” (emotional support). The doctors assessed the importance of these expectations of older patients on a Likert scale from 1 to 7 (1=completely irrelevant, 7=very relevant). Each section score was calculated as a mean value (the sum of item scores divided by the number of items in a given section). The possible section score is between 1 and 7. The higher the score, the more significant the patient’s expectation, as perceived by the GP. Reliability coefficients of PRACTA-PES-D scales were good or very good and ranged from .79 to .96 at time 1 and from .82 to .95 at time 2.

#### GPs’ Self-Assessed Communication Behaviors

We assessed communication behavior with the PRACTA Communication Scale-Doctors (PRACTA-CS-D), comprising 26 items that allowed for calculating a global score or that could be analyzed separately (Multimedia Appendix 3 shows the full scale in English translation). GPs rated the frequency of communicational behavior presented in each item (eg, “During visits of my elderly patients (65+) I…greet them in a kind manner,” “listen to them carefully,” “make sure I understood them correctly,”). The answers were provided on a 7-point Likert scale, where 1=very seldom and 7=very often. The global score was calculated as a mean value of all item scores. It ranged from 1 to 7. The higher the score, the higher the frequency of communication behavior declared by GPs. Reliability coefficients of global scores before and after the intervention were .94 and .95, respectively.

#### GPs’ Perception of Older Patients’ ATH

We evaluated ATH by 2 tools: the PRACTA Attitude Toward Treatment and Health Scale-Doctors (PRACTA-ATH-D) and the PRACTA Self-Efficacy Scale-Doctors (PRACTA-SE-D) (Multimedia Appendix 4 to see all items measuring ATH: PRACTA-SE-D items directly follow PRACTA-ATH-D items). PRACTA-ATH-D comprised 16 items, which formed 4 scales reflecting 4 aspects of attitude: cognitive (6 items), emotional-positive (3 items), emotional-negative (3 items), and motivational (4 items). We developed the structure of this scale based on exploratory and confirmatory factor analyses (unpublished data, 2017). Each item started with the statement “Usually, the elderly patients (65+) after a visit at my office…” followed by statements indicating older patients’ ATH, such as “understand the nature and causes of their ailments,” feel calmer,” “have fears about their symptoms,” “are going to participate in the treatment actively.” GPs responded on a 7-point Likert scale (1=definitely no, 7=definitely yes). Each scale score was calculated as a mean value (the sum of item scores divided by the number of items in a scale). The possible scale score was between 1 and 7. Higher scores suggest a more active attitude in all aspects, except for the negative emotions. Reliability coefficients of PRACTA-ATH-D scales were good or very good and ranged from .88 to .93 before the intervention and from .88 to .94 after the intervention.

PRACTA-SE-D is a unidimensional scale consisting of 3 items that measure GPs’ perception of older patients’ self-efficacy related to health behavior changes: the fifth aspect of ATH included in the study. The statement “Usually, the elderly patients (65+) after a visit at my office…” was followed by items indicating their patients’ sense of self-efficacy, such as “think they can influence how they’ll feel in the future.” Possible responses were from 1=definitely no to 7=definitely yes. The score was calculated as a mean value. Higher scores indicated greater self-efficacy. The reliability coefficients of the PRACTA-SE-D scale at time 1 and time 2 were .90.

### Statistical Analysis

To compare the study groups with respect to descriptive statistics, we used a chi-square test (for frequencies) and analysis of variance (for interval and ratio scales; in cases where the assumption of variance homogeneity for analysis of variance was not met, we applied the Brown-Forsythe correction) [40].

In the final study group, there were no missing values at time 1, and at time 2 they were <0.5%. The Little missing completely at random test pointed to random missingness (*P*=.15).

To take into account possible changes occurring in the measured variables between time 1 and time 2, we calculated the indexes of change for each variable (as the time 2–time 1 difference). The index of change above zero indicated an increase in GPs’ ratings (ie, GPs at time 2 rated older patients’ expectations as more important, the patients’ attitude as more active, and their own communication behavior as more frequent than those at time 1) and the index of change below zero demonstrated a decrease in these ratings between time 1 and time 2. Subsequently, we applied the indexes in the tested models as outcome variables.

In most cases, the Kolmogorov-Smirnov test showed that the outcome variables were not normally distributed (*P*<.05). Thus, to analyze the significance of differences between indexes of change in groups and to test interaction effects, we used the generalized linear model [41]. In the tables below, we present the results of the generalized linear model performed without an intercept (unstandardized B parameters equal to mean values of variables). We used pairwise comparisons with Bonferroni correction for multiple comparisons. We used the same type of analysis to compare the study groups in variables measured at time 1.

We used the following procedure of power calculation. Because our pilot study didn’t have an interventional design, it was impossible to measure the standard deviations of indexes of change of the study variables. We performed a posteriori power calculation for the indexes of change of ATH scales (as key indicators of activation) and used it to verify adequacy of the sample size. We assumed an alpha of .05 and level of power of 80%. The required population size to detect effect sizes defined as a pairwise difference at the level of 0.50 and 0.25 (absolute value) were estimated. The analyses indicated that the size of the groups needed to detect an effect size estimated at 0.50 ranged from 30 to 40 per group depending on the ATH scale, and to detect an effect size estimated at 0.25, the required group size ranged from 118 to 158 per group (the only exception was the negative emotions scale, which needed 107 and 425 participants, respectively).

## Results

### Participants’ Characteristics

Table 1 presents descriptive statistics for the e-learning, pdf article, and control groups. The analyzed groups differed with respect to some features of the facilities. In the e-learning group, there were more doctors working in privately owned facilities and in facilities where times of visits were scheduled individually for every patient. In the e-learning group, there were also fewer doctors working in facilities located in bigger towns (>100,000 residents) and in facilities where the average single visit lasted longer than 15 minutes. Doctors in the e-learning group worked in facilities where the average number of patients assigned to a single doctor was significantly lower than in control group facilities. As for factors concerning the doctors, the total number of working hours per week was significantly lower in the e-learning group than in the pdf article group.

**Table 1:** Descriptive statistics of the study groups.

Characteristics			Group			Test of differences	*P* value
			E-learning (n=42)	Pdf article (n=89)	Control (n=94)		
**Factor describing primary care facilities**							
	**Location (no. of inhabitants), n (%)**						
		<100,000^a^	13 (35)	27 (33)	26 (39)	χ^2^_4_=27.4	<.001
		>100,000	3 (8)	27 (33)	42 (45)		
		Capital city (Warsaw)	21 (57)	27 (33)	14 (15)		
	**Organizational structure of facility, n (%)**						
		State owned	14 (37)	53 (60)	65 (69)	χ^2^_2_=11.7	.003
		Privately owned	24 (63)	36 (40)	29 (31)		
	**Visits system^b^** **, n (%)**						
		Numbers	2 (53)	20 (23)	22 (24)	χ^2^_4_=11.7	.02
		Scheduled time	32 (84)	49 (55)	56 (62)		
		Order of arrival	4 (11)	20 (23)	13 (14)		
	**Average time of visit, n (%)**						
		<15 minutes	17 (49)	27 (31)	22 (26)	χ^2^_2_=6.1	.048
		>15 minutes	18 (51.4)	59 (68.6)	64 (74.4)		
	Average no. of patients per doctor in facility, mean (SD)		1444 (425)	1681 (672)	1754 (791)	B-F_2,215_=3.33^c^	.04
	No. of doctors working in facility, mean (SD)		5.45 (3.12)	4.79 (2.93)	5.41 (3.38)	*F*_2,218_=1.08	.34
**Factors describing doctors**							
	Age in years, mean (SD)		49.56 (11.56)	49.44 (11.35)	50.39 (13.16)	*F*_2,218_=0.15	.86
	**Sex, n (%)**						
		Female	36 (86)	62 (70)	62 (66)	χ^2^_2_=5.7	.06
		Male	6 (14)	27 (30)	32 (34)		
	**Marital status, n (%)**						
		Single	4 (10)	12 (14)	8 (9)	χ^2^_6_=3.2	.79
		Married	33 (79)	65 (73)	77 (82)		
		Divorced/widowed	5 (12)	12 (14)	9 (10)		
	Seniority, mean (SD)		23.90 (12.13)	23.57 (11.99)	23.87 (13.15)	*F*_2,220_=0.02	.98
	Hours weekly in facility, mean (SD)		33.89 (9.48)	32.72 (10.82)	31.34 (9.89)	*F*_2,219_=0.98	.38
	Hours weekly overall, mean (SD)		39.53 (11.01)	45.36 (15.23)	41.54 (13.21)	*F*_2,219_=3.07	.048
	**Training in geriatrics^d^** **, n (%)**						
		None	28 (67)	49 (55)	49 (52)	χ^2^_4_=6.6	.16
		Single	12 (29)	30 (34)	27 (29)		
		Multiple	2 (5)	10 (11)	18 (13)		
	**Percentage of older patients^e^** **, n (%)**						
		<25%	3 (7)	14 (16)	10 (11)	χ^2^_6_=3.7	.72
		25%-50%	19 (45)	32 (36)	33 (35)		
		51%-75%	16 (38)	36 (40)	40 (43)		
		≥75%	4 (10)	7 (8)	11 (12)		
	**Specialization, n (%)**						
		Internal medicine	15 (37)	28 (34)	46 (55)	χ^2^_6_=11.1	.09
		Family medicine	15 (37)	32 (39)	24 (29)		
		2 specializations^f^	9 (22)	13 (16)	9 (11)		
		Others	2 (5)	10 (12)	5 (6)		

^a^This category includes both rural areas and small towns.

^b^Appointment systems were (1) numbers, which informed patients about their place in a queue to see a doctor but not about the time of their appointment (usually it forced patients to come in advance and wait for a long time not to miss their appointment), (2) scheduled time (the patient was informed about the exact time of their appointment), (3) order of arrival (patients were free to choose the time of their appointment but there was no control over patient flow).

^c^B-F: Brown-Forsythe test.

^d^Training in geriatrics encompassed any form of a postgraduate course.

^e^Doctors’ ratings of percentage of older patients (age ≥65 years) among their patients last year.

^f^Two specializations when at least one was internal medicine or family medicine.

We examined differences between the group taking part in the baseline questionnaire and the final study group in terms of all controlled variables concerning facilities and doctors. We found that doctors in the final study group, in comparison with the dropout group, worked in facilities in which there were significantly fewer doctors (*F*_1,467_=14.18, *P*<.001; mean 5.15, SD 3.16 and mean 6.42, SD 3.96, respectively) and doctors working in privately owned facilities dominated in this group (χ^2^_1_=11.7, *P*<.001). Moreover, doctors in the final study group reported a much higher number of working hours in the given facility (Brown-Forsythe test_1,482_=8.68, *P*=.003; mean 32.35, SD 10.20 and mean 29.39, SD 11.84, respectively) and a much lower number of doctors from this group declared multiple training in geriatrics (χ^2^_2_=6.5, *P*=.04).

### Level of Outcome Variables at Time 1

We compared the study groups with respect to initial levels of all variables used to calculate the indexes of change. Significant differences were found in GPs’ perception of older patients’ expectations for treatment explanation (Wald χ^2^=10.6, *P*=.005) and health advice (Wald χ^2^=6.2, *P*=.046). In both cases, the pdf article group scored lower than the control group (*P*=.001 and *P*=.01, respectively). Further differences were found in 3 aspects of ATH: positive emotions (Wald χ^2^=8.1, *P*=.017), negative emotions (Wald χ^2^=9.6, *P*=.008), and motivation (Wald χ^2^=6.2, *P*=.045). The e-learning group scored lower than the control group in perception of older patients’ positive emotions (*P*=.006) and motivation (*P*=.007), and higher in negative emotions (*P*=.004). On the motivational scale, the pdf article group scored lower than the control group as well (*P*=.046). Multimedia Appendix 5 shows trellis plots presenting time 1 and time 2 section and scale scores of each tool for each study group.

### Effects of the PRACTA Intervention on GPs’ Perception of Older Patients’ Expectations

Table 2 presents means, standard errors, and comparative results of indexes of change in the GPs’ perception of older patients’ expectations in each study group, in the form of significance of Wald chi-square tests and pairwise comparisons.

**Table 2:** Indexes of change in general practitioners’ perception of older patients’ expectations by study group.

Variable	Group^a^			Wald χ^2b^ (*P* value)	Pairwise comparisons (*P* value)^c^		
	EL (n=42) mean (SE)	A (n=89) mean (SE)	C n=94) mean (SE)				
					EL–C	A–C	EL–A
**1. Disease explanation**							
	.71 (.165)	.15 (.131)	.01 (.108)	19.7 (<.001)	0.69 (.001)	0.14 (>.99)	0.55 (.03)
**2. Treatment explanation**							
	.33 (.172)	.11 (.121)	–.06 (.092)	4.9 (.18)	0.39 (.14)	0.17 (.83)	0.22 (.86)
**3. Health advice**							
	.23 (.221)	.10 (.136)	–.27 (.128)	6.0 (.11)	0.50 (.15)	0.37 (.15)	0.13 (>.99)
**4. Quality of life**							
	.17 (.323)	.07 (.179)	–.28 (.194)	2.5 (.45)	0.45 (.67)	0.35 (.55)	0.10 (>.99)
**5. Rapport**							
	–.01 (.099)	–.27 (.088)	–.15 (.070)	14.1 (.003)	0.13 (.77)	–0.13 (.76)	0.27^d^ (.04)
**6. Emotional support**							
	–.29 (.140)	–.30 (.121)	.12 (.147)	11.2 (.01)	–0.41 (.13)	–0.42 (.08)	0.01 (>.99)

^a^Study groups were e-learning (EL), pdf article (A), and control (C).

^b^Wald chi-square test of the overall model.

^c^Pairwise comparisons with Bonferroni correction.

^d^Pairwise comparison significant only after use of least squares difference test.

The results presented in Table 2 demonstrate that the greatest differences between the groups regarded the index of change in GPs’ perception of older patients’ expectations for disease explanation. In the e-learning group the importance of this expectation increased more than in the control and pdf article groups. In relation to the GPs’ perception of older patients’ expectations for emotional support and rapport, the overall models also indicated significant main effects of the intervention but with no significant pairwise comparisons. In the case of expectation for emotional support, the pairwise differences only approached significance, indicating that in the pdf article group, the perception of the importance of older patients’ expectations for emotional support decreased in comparison with the control group. In the case of expectations for rapport, applying the least squares difference test (a method less restrictive than the Bonferroni correction) revealed that the index of change in the e-learning group was significantly higher than in the pdf article group (*P*=.04). What was not significantly affected by the intervention was GPs’ perception of older patients’ expectations for treatment explanation, health advice, and quality-of-life improvement.

### Effects of the PRACTA Intervention on GPs’ Self-Assessed Communication With Older Patients

Table 3 presents means, standard errors, and the results of the comparison of indexes of change in communication behavior (global score and specific items) among GPs in each study group, in the form of significance of Wald chi-square tests and pairwise comparisons.

**Table 3:** Indexes of change in general practitioners’ self-assessed communication^a^ by study group.

Scale item	Group^b^			Wald χ^2c^ (*P* value)	Pairwise comparisons (*P* value)^d^		
	EL (n=42) mean (SE)	A (n=89) mean (SE)	C (n=94) mean (SE)				
					EL–C	A–C	EL–A
**Global communication score**							
	.19 (.126)	–.44 (.079)	–.09 (.074)	34.5 (<.001)	0.28 (.18)	–0.35 (.003)	0.63 (<.001)
**1. I greet them in a kind manner.**							
	.05 (.193)	–.21 (.101)	.06 (.110)	4.9 (.18)	–0.01 (>.99)	–0.28 (.19)	0.26 (.69)
**2. I discuss with them the reason for a visit.**							
	.02 (.180)	–.23 (.117)	–.31 (.121)	10.2 (.02)	0.33 (.38)	0.08 (>.99)	0.25 (.74)
**3. I listen to them carefully.**							
	.17 (.190)	–.34 (.094)	–.12 (.096)	15.1 (.002)	0.28 (.55)	–0.22 (.31)	0.50 (.053)
**4. I show understanding of their problems.**							
	.02 (.163)	–.42 (.109)	–.15 (.097)	16.9 (.001)	0.17 (>.99)	–0.27 (.20)	0.44 (.08)
**5. I make sure I understood them correctly.**							
	.14 (.211)	–.35 (.127)	.07 (.134)	8.3 (.04)	0.07 (>.99)	–0.42 (.07)	0.49 (.14)
**6. I encourage them to ask questions.**							
	.62 (.286)	–.18 (.165)	.04 (.168)	5.9 (.11)	0.58 (.25)	–0.22 (>.99)	0.80 (.046)
**7. I answer all their questions.**							
	–.07 (.163)	–.47 (.114)	–.42 (.120)	29.5 (<.001)	0.34 (.27)	–0.06 (>.99)	0.40 (.13)
**8. I make sure they understood me correctly.**							
	.07 (.166)	–.63 (.126)	–.13 (.125)	26.4 (<.001)	0.20 (>.99)	–0.50 (.01)	0.70 (.002)
**9. I use language they can understand.**							
	.00 (.135)	–.56 (.100)	–.11 (.108)	32.3 (<.001)	0.10 (>.99)	–0.48 (.006)	0.56 (.002)
**10. I summarize topics we discussed.**							
	.21 (.204)	–.47 (.133)	–.15 (.131)	15.0 (.002)	0.36 (.40)	–0.32 (.25)	0.67 (.01)
**11. I inform them about the examination.**							
	.07 (.229)	–.27 (.135)	.02 (.165)	4.1 (.25)	0.05 (>.99)	–0.29 (.52)	0.34 (.60)
**12. I care about their comfort during the examination.**							
	.21 (.181)	–.52 (.101)	–.28 (.125)	32.0 (<.001)	0.49 (.08)	–0.24 (.41)	0.73 (.001)
**13. I provide as much time as they need for each part of the visit.**							
	.21 (.281)	–.51 (.183)	–.15 (.165)	9.0 (.03)	0.36 (.80)	–0.35 (.45)	0.72 (.10)
**14. I explain treatment options available in their situation.**							
	.36 (.220)	–.38 (.126)	–.13 (.109)	13.3 (.004)	0.48 (.15)	–0.25 (.38)	0.74 (.01)
**15. I explain why they should comply with the recommendations.**							
	.14 (.174)	–.40 (.109)	–.14 (.107)	16.2 (.001)	0.28 (.50)	–0.27 (.24)	0.55 (.02)
**16. I make sure they’ll be able to comply with the recommendations.**							
	.02 (.167)	–.40 (.134)	.11 (.130)	9.7 (.02)	–0.08 (>.99)	–0.51 (.02)	0.43 (.14)
**17. I write down the main recommendations for them.**							
	–.02 (.145)	–.43 (.103)	–.13 (.088)	19.4 (<.001)	0.10 (>.99)	–0.30 (.08)	0.40 (.07)
**18. I discuss the plan of further treatment.**							
	.29 (.182)	–.40 (.109)	–.06 (.114)	16.6 (.002)	0.35 (.31)	–0.34 (.09)	0.69 (.003)
**19. I briefly summarize the entire visit.**							
	.36 (.222)	–.46 (.140)	.05 (.148)	13.5 (.004)	0.30 (.77)	–0.51 (.04)	0.82 (.006)
**20. I encourage them to participate in making decisions.**							
	.64 (.164)	–.43 (.148)	.05 (.138)	23.8 (<.001)	0.59 (.02)	–0.48 (.053)	1.07 (<.001)
**21. I give the opportunity to express their opinion.**							
	.62 (.168)	–.48 (.145)	–.10 (.132)	25.2 (<.001)	0.71 (.002)	–0.38 (.14)	1.10 (<.001)
**22. I take their opinion into account in making decisions.**							
	.38 (.178)	–.66 (.146)	.11 (.145)	25.9 (<.001)	0.25 (.69)	–0.77 (.001)	1.04 (<.001)
**23. I create an atmosphere that allows them to discuss intimate issues freely.**							
	.21 (.163)	–.48 (.131)	.02 (.127)	16.6 (.001)	0.19 (>.99)	–0.50 (.01)	0.68 (.002)
**24. I notice their feelings and accept them.**							
	.07 (.170)	–.51 (.106)	.03 (.132)	23.2 (<.001)	0.04 (>.99)	–0.54 (.005)	0.58 (.01)
**25. I ensure a good atmosphere during the entire visit.**							
	.10 (.183)	–.66 (.121)	–.23 (.100)	35.8 (<.001)	0.33 (.35)	–0.43 (.02)	0.76 (.002)
**26. I win their trust.**							
	.07 (.156)	–.53 (.119)	–.21 (.116)	23.2 (<.001)	0.28 (.43)	–0.31 (.18)	0.60 (.007)

^a^Assessed by the Promoting Active Aging Communication Scale-Doctors (PRACTA-CS-D) in response to questions about general practitioners’ usual behavior with their older patients (≥65 years).

^b^Study groups were e-learning (EL), pdf article (A), and control (C).

^c^Wald chi-square test of the overall model.

^d^Pairwise comparisons with Bonferroni correction.

As Table 3 shows, the intervention had a significant impact on the global communication score. Importantly, the changes observed in the pdf article group and the e-learning group were in opposite directions, with a decrease in the pdf article group and an increase in the e-learning group. The index of change in the pdf article group was much lower than in the control and e-learning groups.

At the level of specific GPs communication behavior, between-group differences were significant in 20 of 26 analyzed cases (significant in the overall model and in the difference between at least 2 groups). The mean values of the indexes of change in the pdf article group and e-learning group demonstrated that changes were in opposite directions. In 19 cases, in the pdf article group indexes of change in communication with older patients were significantly different from those in the e-learning group, and in most cases (n=12) also in the control group. The most distinct differences between the e-learning and the pdf article groups (with the difference exceeding the level of 1) were in the following behaviors: encouraging older patients to participate in making decisions, giving them the opportunity to express their opinions, and taking their opinions into account in making decisions. It is worth noting that such differences exceeded the level of 0.5 in a further 13 items.

There were no between-group differences in only 5 cases: greeting older patients in a kind manner, discussing the reasons for their visit, answering all their questions, informing them about the examination, and providing as much time as they needed. In case of 1 item (“I encourage them to ask questions”) the overall model was not significant, but a very large positive change in the e-learning group resulted in e-learning having a significant effect compared with the pdf article.

### Effects of the PRACTA Intervention on GPs’ Perception of Older Patients’ ATH at the End of the Visit

Table 4 presents means, standard errors, and comparative results of indexes of change concerning GPs’ perception of older patients’ ATH at the end of the visit, in the form of significance of Wald chi-square tests and pairwise comparisons.

**Table 4:** Indexes of change in general practitioners’ perception of older patients’ attitude toward treatment and health by study group.

Variable	Group^a^			Wald χ^2b^ (*P* value)	Pairwise comparisons (*P* value)^c^		
	EL (n=42) mean (SE)	A (n=89) mean (SE)	C (n=94) mean (SE)				
					EL–C	A–C	EL–A
**1. Cognitive aspect**							
	.26 (.163)	–.13 (.120)	–.11 (.097)	5.02 (.17)	0.37 (.15)	–0.03 (>.99)	0.40 (.15)
**2. Positive emotions**							
	.19 (.145)	–.20 (.102)	–.17 (.098)	8.7 (.03)	0.36 (.11)	–0.02 (>.99)	0.39 (.08)
**3. Negative emotions**							
	–.54 (.267)	–.04 (.196)	–.23 (.190)	5.5 (.14)	–0.31 (>.99)	0.18 (>.99)	–0.50 (.41)
**4. Motivational aspect**							
	.35 (.175)	–.17 (.114)	–.18 (.107)	8.9 (.03)	0.53 (.03)	0.01 (>.99)	0.52 (.04)
**5. Self-efficacy**							
	.35 (.165)	.01 (.129)	–.05 (.104)	4.9 (.17)	0.41 (.11)	0.06 (>.99)	0.35 (.29)

^a^Study groups were e-learning (EL), pdf article (A), and control (C).

^b^Wald chi-square test of model effect.

^c^Pairwise comparison with Bonferroni correction.

The strongest between-group effects occurred in relation to changes in GPs’ perception of older patients’ motivation for active participation. There were significant differences between the e-learning group and the control and pdf article groups. Similar but weaker effect occurred in relation to GPs’ perception of positive emotions demonstrated by older patients at the end of the visit. The difference between the e-learning and pdf article groups only approached significance. The intervention did not significantly affect GPs’ perception of the following aspects of ATH: cognitive, negative emotions, and self-efficacy.

### Moderators of Intervention Effects

We found effects of moderation in 2 organizational factors: the number of patients assigned to a single GP and facility’s organizational structure.

We found group × number of patients per GP interactions in the following types of expectations: disease explanation (Wald χ^2^=15.7, *P*<.001), treatment explanation (Wald χ^2^=12.9, *P*=.002), quality-of-life improvement (Wald χ^2^=13.1, *P*=.001), and health advice (Wald χ^2^=5.7, *P*=.06). In the e-learning group, in contrast to the other groups, there was a positive relationship between the number of patients per GP and the GPs’ perception of older patients’ expectations for disease explanation. An increase in the number of patients per GP by 1000 increased the importance of older patients’ expectations for disease explanation by an average of 0.95 (B=0.95, *P*=.04) and increased treatment explanation expectation by 1.05 (B=1.05, *P*=.001). At the same time, in the case of quality-of-life improvement, an increase in the number of patients per GP by 1000 resulted in a decrease in the outcome by 2.01 (B= –2.01, *P*=.01) in the e-learning group and by 1.38 (B= –1.38, *P*=.001) in the pdf article group. In the pdf article group, along with the increasing number of patients per GP by 1000, the perception of the importance of older patients’ expectation for health advice decreased by 0.86 (B= –0.86, *P*=.02). We also found group × number of patients per GP interactions in relation to GPs’ perception of 2 aspects of older patients’ ATH: negative emotions (Wald χ^2^=27.5, *P*<.001) and self-efficacy (Wald χ^2^=9.0, *P*=.01). Contrary to the other groups, in the e-learning group, together with the increase by 1000 of the number of patients per GP, negative emotions declined by 3.2 (B= –3.2, *P*<.001) and perception of older patients’ self-efficacy increased by 1.33 (B=1.33, *P*=.004).

Facility organizational structure differentiated the effects of the intervention in terms of GPs’ perception of older patients’ motivation for active participation (Wald χ^2^=10.8, *P*=.004) and their self-efficacy (at the level of statistical trend: Wald χ^2^=5.4, *P*=.07). Doctors in the e-learning group working in privately owned facilities reported an increase in older patients’ motivation by 1.13 (B=1.13, *P*=.005) and in their self-efficacy by 0.85 (B=0.85, *P*=.02), whereas the changes in other intervention groups and type of facility were not significant. In the case of positive emotions, the effect of interaction was not statistically significant (Wald χ^2^=4.6, *P*=.10), but in the e-learning group, doctors working in privately owned facilities scored higher by 0.77 (B=0.77, *P*=.04) than those in other the groups. Moreover, the effect of the statistical trend occurred in relation to GP’s perception of the importance of older patients’ expectations for disease explanation (Wald χ^2^=5.8, *P*=.05). Doctors in the e-learning group working in privately owned facilities reported an increase in perception of such older patient needs by 0.83 (B=0.83, *P*=.02) compared with the other groups.

## Discussion

### Principal Results and Comparison With Prior Work

#### GPs’ Perception of Older Patients’ Expectations

The strongest effect in changes in GPs’ perception of older patients’ expectations for the medical encounter occurred in the e-learning group, in which there was a larger increase in their perception of the importance of explaining the patient’s disease than in the control and pdf article groups. There were similar, though not statistically significant, increases in expectations for health advice and treatment explanation. Doctors in the e-learning group were more likely after than before the intervention to state that such needs were important to older patients. Such changes may help to create an image of older patients free from negative stereotypes [30]. For instance, the stereotype that older age means reduced cognitive abilities, including understanding and appropriate use of information about the disease, can lead to infantilization of the elderly, often manifested in the form of elderspeak [42] and moralizing attitudes about older patients [43]. The intervention had no effect on expectations for quality-of-life improvement. The question arises as to how strongly doctors believe that dealing with this aspect of older patients’ lives belongs to the competence of a doctor [44].

Although the main effects of the intervention regarding expectations for rapport and emotional support were significant, differences between groups only approached significance. In the pdf article group, the doctors after completing the intervention stated that expectations for emotional support and rapport were not as important for older patients as they had thought before the intervention. These changes could have been due to the doctors’ increased awareness of older patients’ needs and, in consequence, to relativization of the meaning of the expectation for rapport and emotional support. There is a fairly common belief among physicians that older patients visit their GP solely for social reasons [31]. As a result, doctors may overestimate the importance of older patients’ emotional and relational needs and focus less on the need for information and medical care [24].

Generally, the main impact of e-learning was the growing importance of older patients’ cognitive expectations, especially their expectations for disease explanation, in comparison with the pdf article group. This would allow for only partial confirmation of hypothesis 1a. We formulated no directional hypotheses regarding expectations for rapport and social support, but the decrease we observed in the pdf article group appears to be slightly larger than that in the e-learning group.

#### GPs’ Self-Assessed Communication With Older Patients

In terms of self-assessed communication, the 2 forms of intervention yielded different effects, with a substantial decrease in the pdf article group and a moderate increase in the e-learning group. Doctors in the pdf article group less frequently behaved in a desired manner after the intervention, both at the level of the global score and at the level of 20 of 26 specific behaviors. Desired GP behavior seems unlikely to have decreased as a result of the intervention. What might be likely, on the other hand, is that their assessment of their own behavior changed, as compared with the behavior described in the pdf article; doctors might have realized how their behavior differed from what was the most desirable. This would demonstrate doctors’ growing self-criticism on reading the pdf article. It is worth noting that only 5 of the 26 described behaviors were not affected by the pdf article at all. Paradoxically, although the direction of change was negative, the intervention achieved the desired effect. In the e-learning group, 2 types of behavior increased in frequency. They both concerned actions aimed at activating older patients. This means that the pdf article contributed to GPs’ critical self-assessment of their own communication behavior, but that e-learning would have a greater potential to enhance skills or GPs’ subjective sense of their improved skills [34]. Although in the majority of cases the increase in self-assessed communication in the e-learning group was not significantly larger than in the control group, it was larger than in the pdf article group. We did not hypothesize that we would see such a decrease in self-assessed communication in the pdf article group, but hypothesis 1b may be partially confirmed.

#### GPs’ Perception of Older Patients’ ATH

The strongest effect on ATH was in the e-learning group, in GPs’ perception of older patients’ motivation to actively participate was significantly greater than in the control and pdf article groups. The second important result was the rise, compared with the pdf article group, in the level of positive emotions observed in older patients. Both aspects are the essence of successful aging [36]. It is worth noting that, contrary to the pdf article and control groups, in the e-learning group changes were positive in all aspects of ATH (apart from negative emotions). The results regarding changes in doctors’ perception of older patients’ attitudes as a result of participating in e-learning are consistent with hypothesis 1c, but the pdf article did not have any significant effect in this area.

#### Moderators of Intervention Effects

Among numerous factors considered as potential moderators of the intervention, only 2 organizational factors proved to be significant. In the e-learning group, having more patients assigned to the doctor increased the importance of expectations for disease and treatment explanation, increased the level of patient self-efficacy, and lowered the intensity of their negative emotions as perceived by GPs. This might mean that doctors with a greater number of patients achieved more of the desired effects of e-learning.

However, in both intervention groups, having more patients reduced the importance of expectations for quality-of-life improvement. Numerous factors are associated with the number of patients assigned to a doctor, and further research is required to determine which of them contributed most to the results. One can only speculate that, for example, higher remuneration and job satisfaction of doctors with a greater number of patients would foster greater involvement and motivation to expand their competences; on the other hand, more duties would result in cutting down the appointment time, reducing the focus on the patient, and disregarding their needs for improving quality of life.

Another factor that moderated the effects of the intervention was the facility organizational structure. Among doctors in the e-learning group working in privately owned facilities, the effect concerning perception of older patients as more motivated to actively participate was significantly higher than in the other groups. Furthermore, as shown by other effects, although weaker, it was this group who benefited from this type of intervention more than the others. This suggests that privately owned facilities create better conditions for e-learning to be effective, as they offer better organizational conditions, or GPs working in or running private medical services are more likely to have psychosocial dispositions such as openness to new experience or flexibility. Generally, the results confirm the validity of studies looking for potential moderators of intervention effects [33].

### Limitations

This study had selection bias [32]. Although recruitment of facilities for the study was random, only one-fifth of managers approved and then only half of the invited doctors took part in the assessment at time 1. One can speculate that such approval was given only by managers who were not afraid of any evaluation and who welcomed research and innovations. Despite random allocation of facilities to the intervention, only some doctors participated in the interventions and the assessment at time 2 (attrition bias). More GPs in the final group than in the dropout group worked in privately owned facilities and in facilities employing a lower number of doctors. Moreover, doctors from these facilities declared more working hours in a given facility, but less training in geriatrics. This may indicate that GPs who had agreed to participate in the program enjoyed a better organizational setting and might be more dedicated to their facilities. They also seemed to be good prospects for training in older patient activation.

As a result of attrition, the study groups sizes were not fully satisfactory in relation to the results of power sample calculations (as they were insufficient to detect smaller differences). Moreover, the study groups were not balanced at entry, in terms of both some descriptive statistics and selected study variables. In the e-learning group more GPs worked in privately owned facilities located in the capital or small towns or villages, with more patient-customized appointment systems (scheduled individually) and fewer patients assigned to a single GP. These features seemed to be conducive to participation in the intervention, but in this study they did not turn out to be sufficient. As for outcome variables at time 1, the e-learning group scored lower than the control group in perception of older patients’ positive emotions and motivation, and higher in perception of negative emotions. These differences might partially explain why we observed significant effects of e-learning in some of these aspects of ATH. On the other hand, the pdf article group scored lower than the control group in perception of older patients’ expectations for treatment explanation and health advice, but with no impact on the effects of the pdf article in such areas.

However, the described recruitment process reflects the actual situation, and confirms extreme difficulties in conducting such a study under natural conditions and in implementing the intervention; these limitations were also reported in other studies [32-34]. This means that the results of this study should be interpreted with caution and can’t be generalized to the entire population of GPs. Apart from the above-described limitations, there were also specific inclusion criteria, such as having a contract signed with the National Health Fund and the location in central Poland. The first criterion suggests that the results apply only to patients who did not cover the cost of medical service themselves, whereas the location criterion suggests that any conclusions should be restricted to GPs and older patients living in wealthier areas of the country.

Generally, the study group seemed to demonstrate specific features that might have been responsible for relatively small effect sizes of the intervention, as there were also effects of statistical trends in addition to statistically significant effects. As mentioned above, the sample sizes of the study groups were insufficient to detect smaller differences, and only those above the level of 0.5 were marked as significant.

It should be noted that all outcome variables were subjective. They encompassed GPs’ perception of older patients and GPs’ perception of their own performance; no performance, however, was objectively measured. Thus, the analysis of older patients’ attitudes in the context of doctors’ attitude change would be a valuable complement to the results.

A separate analysis is needed to discover the reasons for such a low proportion of doctors in e-learning. The reasons can be divided into 2 groups depending on the time of making the decision: before or after entering the intervention. As for reasons, which might matter before the start, the e-learning requirement to use more advanced technology seems to be a disincentive. There was no age difference between the study groups. The participants’ average age was about 50 years. Thus, low computer literacy and a fear of advanced technology can’t be ruled out as deterrents to starting the intervention. Participation in the pdf article intervention, where the use of a computer was only required to read the article, was higher. Of 208 doctors in the pdf article and control groups who received the opportunity to participate in PRACTA e-learning at the end of the time 2 assessment, 117 declared a desire to participate but ultimately only 7 doctors logged on to the platform. Perhaps GPs would like to participate in such training but reasons such as lack of time, fatigue, and the need to focus on urgent tasks prevent them from participating.

On the other hand, 11 (21%) of 53 GPs who only logged on and a further 10 (19%) who completed no more than 3 modules decided to drop out of the study after starting the e-learning intervention. The question arises as to whether the course in the form of a multimedia game demanding active participation from the learner was adequate for such a group of GPs. Additionally, each module took about an hour to complete, and the range of subjects and cognitive load could have been excessive for some GPs. At the same time, the majority of GPs who had started the e-learning (71%) completed all 5 modules.

Another important issue was the extent of correspondence between the e-learning and pdf article interventions. We assumed that both forms of the intervention were identical in terms of messages directed to learners but were distinct from each other in their delivery methods. The main objective of the pdf article was to describe active aging and GPs’ ways of enhancing active aging, whereas the objective of the e-learning was not only to describe it but also to demonstrate (to model) how to do it. Based on theories of social learning, knowledge is important in changing attitudes and behavior, but is insufficient on its own. Only combined with training and creating new habits does knowledge increase the amplitude of such change [38]. Therefore, after having read the pdf article, GPs were expected to know what to do (what is recommended), but they did not receive any training in how to do it (as a consequence, the change in outcomes was smaller), whereas after e-learning they were expected to be equipped not only with knowledge but also with skills (as a consequence, the change in outcomes was larger). However, it is very likely that the methods used in the e-learning (eg, case study, video) might at least slightly have changed the message contained in the text material (number of details, associations, practical implications). Therefore, it seems of great importance to precede implementation of the intervention with a pilot study to verify content consistency of both forms and to correct any discrepancies. Doing so would give deeper insights into factors responsible for the achieved results.

The above limitations clearly demonstrate the need to replicate the study in larger groups. Further research is required to verify content consistency of both forms of the intervention, but also the relationships between the studied variables and their impact on the achieved effects of the intervention. A valuable complement to the results would be to analyze the patients’ attitudes in the context of doctors’ behavioral change. This will be the next step in our ongoing project.

### Conclusions

As far as expectations are concerned, the main impact of e-learning was the growing importance of older patients’ cognitive expectations, especially the expectation for disease explanation. In terms of self-assessed communication, the 2 forms of intervention yielded different effects, with a substantial decrease in the pdf article group and a moderate increase in the e-learning group. Despite the negative direction of changes in the pdf article group, such a result may be perceived as a desired effect of the intervention, as it reflects a critical self-assessment by doctors of their own communication behavior. In terms of the ATH, the strongest effect concerned the e-learning group, in which there was a significant increase in GPs’ perception of older patients’ motivation for active participation and positive emotions compared with the control and pdf article groups. Among many factors moderating the effects of intervention, 2 factors of organizational character proved to be significant: the number of patients assigned to a single GP and the facility organizational structure. In the e-learning group, having more patients assigned to the doctor increased the importance of expectations for disease and treatment explanation, and of perceiving higher levels of self-efficacy and a lower intensity of negative emotions among older patients. In both intervention groups, having more patients reduced the importance of the expectation for quality-of-life improvement. In the e-learning group of doctors working in privately owned facilities, the effect of perceiving older patients as being more motivated to actively participate was significantly higher than in the other groups.

The results demonstrate the suitability of the 2 methods, but in other areas and under different conditions. The key benefit of the pdf article intervention was doctors’ growing reflection on their limitations in terms of communication skills, whereas e-learning was more effective in changing the perception of older patients’ proactive attitude, especially among GPs working in privately owned facilities and having a higher number of assigned patients. Although we did not achieve all the expected effects of the PRACTA intervention, both its forms seem promising in terms of growing competencies of GPs in communication with and activation older patients.

## Appendix

Multimedia Appendix 1 Contents of the Promoting Active Aging (PRACTA) intervention.

Multimedia Appendix 2 Promoting Active Aging (PRACTA) Patients Expectations Scale - Doctors (PRACTA-PES-D).

Multimedia Appendix 3 Promoting Active Aging (PRACTA) Communication Scale - Doctors (PRACTA-CS-D).

Multimedia Appendix 4 Promoting Active Aging (PRACTA) Attitude Toward Treatment and Health Scale - Doctors (PRACTA-ATH-D) and PRACTA Self-Efficacy Scale - Doctors (PRACTA-SE-D).

Multimedia Appendix 5 Trellis plots of time 1 and time 2 section and scale scores for e-learning, pdf article, and control groups.

## Abbreviations

ATH: attitude toward treatment and health
GP: general practitioner
PRACTA: Promoting Active Aging
PRACTA-ATH-D: Promoting Active Aging Attitude Toward Treatment and Health Scale-Doctors
PRACTA-CS-D: Promoting Active Aging Communication Scale-Doctors
PRACTA-PES-D: Promoting Active Aging Patients Expectations Scale-Doctors
PRACTA-SE-D: Promoting Active Aging Self-Efficacy Scale-Doctors
RQ: research question
