# Supplementary material for: Enhancing Doctors’ Competencies in Communication With and Activation of Older Patients: The Promoting Active Aging (PRACTA) Computer-Based Intervention Study
Source: J Med Internet Res. 2017 Feb 22;19(2):e45. doi: 10.2196/jmir.6948 (PMC5343213; doi:10.2196/jmir.6948)
Supplement: Multimedia Appendix 1 [file jmir_v19i2e45_app1.pdf]

Pdf article	E-learning	
Text and images only	The same content as in pdf article but supported by other methods, extended with additional activities	
**Subjects**	**Methods specific for e-learning**	**Missions[e]**
1. Process of active aging and the importance of an active attitude toward health		
Active and successful aging	Presentation guided by a user + quiz[b]<br>Mini-lecture + quiz	Study a problem<br>Go for a lecture
Opportunities of resources allocation in older patients	Presentation in a form of interview<br>Case study + quiz	Meet a WHO expert
Criteria of an active attitude toward health	Case study + quiz<br>Mini-lecture	Go to GP's office to meet an active older patient
2. Doctors' beliefs about older adults' abilities and expectations		
ABC model and older adults' activity	A game + self-assessment + quiz	Perform a street interview
Stereotypes about the elderly and manifestation of ageism	Presentation guided by a user + quiz<br>Case study + quiz	Go for a meeting with older patients<br>Prepare a poster
Older patients' expectations in GP office	Presentation guided by a user[c] + quiz<br>Mini-lecture + quiz	Participate in a conference
3. The importance of physician-patient rapport for older patients and health outcomes		
Types of GP-patient rapport	Case studies<br>Presentation guided by a user + quiz	Meet the doctor who investigates older patients' complaints
Technics and rules of communication	Animated cartoon + quiz<br>Presentation guided by a user + quiz	Prepare a guideline on how to proceed
Strategies of dealing with older patients' emotional needs	Mini-lecture + quiz<br>A video[d] demonstrating technics presented in the module	Meet an expert<br>Prepare a video
4. Psychological rules and skills for promoting an active attitude toward health		
Models of health behavior change	A puzzle<br>Mini-lecture + quiz	Go to a lab and meet experts
Technics – higher level of older patient's motivation	A video demonstrating new skills<br>Analysis of technics + quiz	Join a lab team to solve problems
Technics – lower level of older patient's motivation	A video demonstrating new skills<br>Analysis of technics + quiz	Join a lab team to solve problems
5. Quality of life and providing support for older patients		
Aspects of quality of life	Presentation guided by a user + quiz<br>Mini-lecture + quiz	Participate in a teleconference
GPs' role in improving older patients' quality of life	A video demonstrating new skills<br>Analysis of technics + quiz	Meet experts and watch a video
GPs as a source of social support for older patients	Presentation guided by a user + quiz<br>Animated cartoon + quiz	Meet experts to solve a problem

[a] the subjects were the same for both forms of intervention

[b] quiz assumed repetition of knowledge or a technic previously presented, its completion allowed to receive a trophy which served as a mnemonic and an award in the game

[c] it assumed that the user decided on the order of subjects' selection

[d] each video presented 8-10 minute GP-older patient conversation and was recorded on the purpose of the project

[e] e-learning was designed as a story. Each module began with a meeting with a Master (unidentified very important person) who ordered missions and ended with a report summarizing a module (it was arranged as another task performed by a user).
