# Supplementary material for: Enhancing Doctors’ Competencies in Communication With and Activation of Older Patients: The Promoting Active Aging (PRACTA) Computer-Based Intervention Study
Source: J Med Internet Res. 2017 Feb 22;19(2):e45. doi: 10.2196/jmir.6948 (PMC5343213; doi:10.2196/jmir.6948)
Supplement: Multimedia Appendix 2 [file jmir_v19i2e45_app2.pdf]

# PRACTA-PES-D

Below, you will find a list of statements referring to various elements of a visit at a doctor's office. Under each statement there is a 7-point response scale. **Please mark with an X the number that describes best, how important is a particular element of the visit for your elderly patients (65+)**. Number 1 indicates that it is completely irrelevant, and number 7 – that it is very relevant. Please make sure you answered all questions.

**Usually, during a visit elderly patients (65+) expect me …**

1. … to find the cause of their symptoms.

1 ☐ ——— 2 ☐ ——— 3 ☐ ——— 4 ☐ ——— 5 ☐ ——— 6 ☐ ——— 7 ☐

***Completely irrelevant*** ***Very relevant***

2. … to present them the probable course of their treatment.

1 ☐ ——— 2 ☐ ——— 3 ☐ ——— 4 ☐ ——— 5 ☐ ——— 6 ☐ ——— 7 ☐

***Completely irrelevant*** ***Very relevant***

3. … to discuss possible consequences of illness.

1 ☐ ——— 2 ☐ ——— 3 ☐ ——— 4 ☐ ——— 5 ☐ ——— 6 ☐ ——— 7 ☐

***Completely irrelevant*** ***Very relevant***

**Usually, during a visit the elderly patients (65+) expect me …**

1. … to present the results of the tests performed.

1 ☐ ——— 2 ☐ ——— 3 ☐ ——— 4 ☐ ——— 5 ☐ ——— 6 ☐ ——— 7 ☐

***Completely irrelevant*** ***Very relevant***

2. … to give them advice about medications they take.

1 ☐ ——— 2 ☐ ——— 3 ☐ ——— 4 ☐ ——— 5 ☐ ——— 6 ☐ ——— 7 ☐

***Completely irrelevant*** ***Very relevant***

3. … to present recommendations for their treatment.

1 ☐ ——— 2 ☐ ——— 3 ☐ ——— 4 ☐ ——— 5 ☐ ——— 6 ☐ ——— 7 ☐

***Completely irrelevant*** ***Very relevant***

**Usually, during a visit the elderly patients (65+) expect me …**

1. … to talk to them about how they feel and how they cope.

1 ☐ ——— 2 ☐ ——— 3 ☐ ——— 4 ☐ ——— 5 ☐ ——— 6 ☐ ——— 7 ☐

***Completely irrelevant*** ***Very relevant***

2. … to give them reassurance.

1 ☐ ——— 2 ☐ ——— 3 ☐ ——— 4 ☐ ——— 5 ☐ ——— 6 ☐ ——— 7 ☐

***Completely irrelevant*** ***Very relevant***

3. … to show them my care.

1 ☐ ——— 2 ☐ ——— 3 ☐ ——— 4 ☐ ——— 5 ☐ ——— 6 ☐ ——— 7 ☐

***Completely irrelevant*** ***very relevant***

Translation only

© PRACTA Team, WUM/NTNU

**Usually, during a visit the elderly patients (65+) expect me …**

1. … to talk to them about what harms their health.

1 ☐ ——— 2 ☐ ——— 3 ☐ ——— 4 ☐ ——— 5 ☐ ——— 6 ☐ ——— 7 ☐

***Completely irrelevant*** ***Very relevant***

2. … to advise them what they can do to improve their functioning in everyday life.

1 ☐ ——— 2 ☐ ——— 3 ☐ ——— 4 ☐ ——— 5 ☐ ——— 6 ☐ ——— 7 ☐

***Completely irrelevant*** ***Very relevant***

3. … to encourage them to make health promoting changes (physical activity, diet).

1 ☐ ——— 2 ☐ ——— 3 ☐ ——— 4 ☐ ——— 5 ☐ ——— 6 ☐ ——— 7 ☐

***Completely irrelevant*** ***Very relevant***

**Usually, during a visit the elderly patients (65+) expect me …**

1. … to suggest how to maintain social relationships.

1 ☐ ——— 2 ☐ ——— 3 ☐ ——— 4 ☐ ——— 5 ☐ ——— 6 ☐ ——— 7 ☐

***Completely irrelevant*** ***Very relevant***

2. … to talk with them how to spend time actively.

1 ☐ ——— 2 ☐ ——— 3 ☐ ——— 4 ☐ ——— 5 ☐ ——— 6 ☐ ——— 7 ☐

***Completely irrelevant*** ***Very relevant***

3. … to suggest ways of maintaining life satisfaction.

1 ☐ ——— 2 ☐ ——— 3 ☐ ——— 4 ☐ ——— 5 ☐ ——— 6 ☐ ——— 7 ☐

***Completely irrelevant*** ***Very relevant***

**Usually, during a visit the elderly patients (65+) expect me …**

1. … to be benevolent towards them

1 ☐ ——— 2 ☐ ——— 3 ☐ ——— 4 ☐ ——— 5 ☐ ——— 6 ☐ ——— 7 ☐

***Completely irrelevant*** ***Very relevant***

2. … to treat them seriously.

1 ☐ ——— 2 ☐ ——— 3 ☐ ——— 4 ☐ ——— 5 ☐ ——— 6 ☐ ——— 7 ☐

***Completely irrelevant*** ***Very relevant***

3. … to show them respect.

1 ☐ ——— 2 ☐ ——— 3 ☐ ——— 4 ☐ ——— 5 ☐ ——— 6 ☐ ——— 7 ☐

***Completely irrelevant*** ***Very relevant***

Translation only

© PRACTA Team, WUM/NTNU
