# Supplementary material for: Enhancing Doctors’ Competencies in Communication With and Activation of Older Patients: The Promoting Active Aging (PRACTA) Computer-Based Intervention Study
Source: J Med Internet Res. 2017 Feb 22;19(2):e45. doi: 10.2196/jmir.6948 (PMC5343213; doi:10.2196/jmir.6948)
Supplement: Multimedia Appendix 3 [file jmir_v19i2e45_app3.pdf]

PRACTA-CS-D

Below you'll find a list of various behaviors of a doctor during a visit and a 7-point response scale. **Mark with an X the number that is the closest to how you usually behave with your elderly patients (65+).** We ask for honest answers.

	**During visits of my elderly patients (65+) I…**	***Very seldom*** … ***Very often***
1.	… greet them in a kind manner.	1☐ ---- 2☐ ---- 3☐ ---- 4☐ ---- 5☐ ---- 6☐ ---- 7☐
2.	… discuss with them the reason for a visit.	1☐ ---- 2☐ ---- 3☐ ---- 4☐ ---- 5☐ ---- 6☐ ---- 7☐
3.	… listen to them carefully.	1☐ ---- 2☐ ---- 3☐ ---- 4☐ ---- 5☐ ---- 6☐ ---- 7☐
4.	… show understanding of their problems.	1☐ ---- 2☐ ---- 3☐ ---- 4☐ ---- 5☐ ---- 6☐ ---- 7☐
5.	… make sure I understood them correctly.	1☐ ---- 2☐ ---- 3☐ ---- 4☐ ---- 5☐ ---- 6☐ ---- 7☐
6.	… encourage them to ask questions.	1☐ ---- 2☐ ---- 3☐ ---- 4☐ ---- 5☐ ---- 6☐ ---- 7☐
7.	… answer all their questions.	1☐ ---- 2☐ ---- 3☐ ---- 4☐ ---- 5☐ ---- 6☐ ---- 7☐
8.	… make sure they understood me correctly.	1☐ ---- 2☐ ---- 3☐ ---- 4☐ ---- 5☐ ---- 6☐ ---- 7☐
9.	… use language they can understand.	1☐ ---- 2☐ ---- 3☐ ---- 4☐ ---- 5☐ ---- 6☐ ---- 7☐
10.	… summarize topics we've discussed.	1☐ ---- 2☐ ---- 3☐ ---- 4☐ ---- 5☐ ---- 6☐ ---- 7☐

	**During visits of my elderly patients (65+) I…**	***Very seldom*** … ***Very often***
11.	… inform them about the examination.	1☐ ---- 2☐ ---- 3☐ ---- 4☐ ---- 5☐ ---- 6☐ ---- 7☐
12.	… care about their comfort during the examination (i.e. measuring blood pressure).	1☐ ---- 2☐ ---- 3☐ ---- 4☐ ---- 5☐ ---- 6☐ ---- 7☐
13.	… provide as much time as they need for each part of the visit (the interview, preparation for the examination, thinking, etc.).	1☐ ---- 2☐ ---- 3☐ ---- 4☐ ---- 5☐ ---- 6☐ ---- 7☐

	**During visits of my elderly patients (65+) I…**	***Very seldom*** … ***Very often***

Translation only

© PRACTA Team, WUM/NTNU

14.	… explain treatment options available in their situation	1☐ ---- 2☐ ---- 3☐ ---- 4☐ ---- 5☐ ---- 6☐ ---- 7☐
15.	… explain why they should comply with the recommendations.	1☐ ---- 2☐ ---- 3☐ ---- 4☐ ---- 5☐ ---- 6☐ ---- 7☐
16.	… make sure they'll be able to comply with the recommendations.	1☐ ---- 2☐ ---- 3☐ ---- 4☐ ---- 5☐ ---- 6☐ ---- 7☐
17.	… write down the main recommendations for them (medications, dosage, etc).	1☐ ---- 2☐ ---- 3☐ ---- 4☐ ---- 5☐ ---- 6☐ ---- 7☐
18.	… discuss the plan of further treatment.	1☐ ---- 2☐ ---- 3☐ ---- 4☐ ---- 5☐ ---- 6☐ ---- 7☐
19.	...briefly summarize the entire visit.	1☐ ---- 2☐ ---- 3☐ ---- 4☐ ---- 5☐ ---- 6☐ ---- 7☐

	**During visits of my elderly patients (65+) I…**	***Very seldom*** ... ***Very often***
20..	… encourage them to participate in making decisions.	1☐ ---- 2☐ ---- 3☐ ---- 4☐ ---- 5☐ ---- 6☐ ---- 7☐
21.	… give the opportunity to express their opinion.	1☐ ---- 2☐ ---- 3☐ ---- 4☐ ---- 5☐ ---- 6☐ ---- 7☐
22..	… take their opinion into account in making decisions.	1☐ ---- 2☐ ---- 3☐ ---- 4☐ ---- 5☐ ---- 6☐ ---- 7☐
23.	… create an atmosphere that allows them to discuss intimate issues freely.	1☐ ---- 2☐ ---- 3☐ ---- 4☐ ---- 5☐ ---- 6☐ ---- 7☐
24.	… notice their feelings and accept them.	1☐ ---- 2☐ ---- 3☐ ---- 4☐ ---- 5☐ ---- 6☐ ---- 7☐
25.	… ensure a good atmosphere during the entire visit.	1☐ ---- 2☐ ---- 3☐ ---- 4☐ ---- 5☐ ---- 6☐ ---- 7☐
26.	… win their trust.	1☐ ---- 2☐ ---- 3☐ ---- 4☐ ---- 5☐ ---- 6☐ ---- 7☐
		Very seldom ... ***Very often***

Translation only

© PRACTA Team, WUM/NTNU
