# Supplementary material for: Enhancing Doctors’ Competencies in Communication With and Activation of Older Patients: The Promoting Active Aging (PRACTA) Computer-Based Intervention Study
Source: J Med Internet Res. 2017 Feb 22;19(2):e45. doi: 10.2196/jmir.6948 (PMC5343213; doi:10.2196/jmir.6948)
Supplement: Multimedia Appendix 4 [file jmir_v19i2e45_app4.pdf]

PRACTA-ATH-D

Statements describing various beliefs and feelings related to a visit at a doctor's office are presented below. Please estimate how true are these beliefs and feelings for the elderly patients (65+) who leave your office. **Mark with an X the box by the number from 1 to 7 that describes your response best for each of the statements.**

Usually, the elderly patients (65+) after a visit at my office…

1. …understand the nature and cause of their ailments.

1☐ ------- 2☐ ------- 3☐ ------- 4☐ ------- 5☐ ------- 6☐ ------- 7☐

definitely no *definitely yes*

2. … know available treatment options for their ailments.

1☐ ------- 2☐ ------- 3☐ ------- 4☐ ------- 5☐ ------- 6☐ ------- 7☐

definitely no *definitely yes*

3. … understand the results of all thier medical tests.

1☐ ------- 2☐ ------- 3☐ ------- 4☐ ------- 5☐ ------- 6☐ ------- 7☐

definitely no *definitely yes*

4. … know how each of their medications works.

1☐ ------- 2☐ ------- 3☐ ------- 4☐ ------- 5☐ ------- 6☐ ------- 7☐

definitely no *definitely yes*

5. … know how to prevent further problems with health.

1☐ ------- 2☐ ------- 3☐ ------- 4☐ ------- 5☐ ------- 6☐ ------- 7☐

definitely no *definitely yes*

6. … know how to sustain or improve health according to their age.

1☐ ------- 2☐ ------- 3☐ ------- 4☐ ------- 5☐ ------- 6☐ ------- 7☐

definitely no *definitely yes*

Usually, the elderly patients (65+) after a visit at my office…

1. … feel calmer.

1☐ ------- 2☐ ------- 3☐ ------- 4☐ ------- 5☐ ------- 6☐ ------- 7☐

definitely no *definitely yes*

2. … believe the treatment will be effective.

1☐ ------- 2☐ ------- 3☐ ------- 4☐ ------- 5☐ ------- 6☐ ------- 7☐

definitely no *definitely yes*

3. … hope their health will improve.

1☐ ------- 2☐ ------- 3☐ ------- 4☐ ------- 5☐ ------- 6☐ ------- 7☐

definitely no *definitely yes*

Translation only

© PRACTA Team, WUM/NTNU

Usually, the elderly patients (65+) after a visit at my office…

1. … feel depressed.

   1☐ ------- 2☐ ------- 3☐ ------- 4☐ ------- 5☐ ------- 6☐ ------- 7☐

   definitely no *definitely yes*

2. … have fears about their symptoms.

   1☐ ------- 2☐ ------- 3☐ ------- 4☐ ------- 5☐ ------- 6☐ ------- 7☐

   definitely no *definitely yes*

3. … doubt that improvement in their functioning is possible.

   1☐ ------- 2☐ ------- 3☐ ------- 4☐ ------- 5☐ ------- 6☐ ------- 7☐

   definitely no *definitely yes*

Usually, the elderly patients (65+) after a visit at my office…

1. … are going to comply to the recommendations conscientiously.

   1☐ ------- 2☐ ------- 3☐ ------- 4☐ ------- 5☐ ------- 6☐ ------- 7☐

   definitely no *definitely yes*

2. … are going to find out more about their health.

   1☐ ------- 2☐ ------- 3☐ ------- 4☐ ------- 5☐ ------- 6☐ ------- 7☐

   definitely no *definitely yes*

3. … are making a plan how to comply to the recommendations.

   1☐ ------- 2☐ ------- 3☐ ------- 4☐ ------- 5☐ ------- 6☐ ------- 7☐

   definitely no *definitely yes*

4. … are going to participate in the treatment actively.

   1☐ ------- 2☐ ------- 3☐ ------- 4☐ ------- 5☐ ------- 6☐ ------- 7☐

   definitely no *definitely yes*

Usually, the elderly patients (65+) after a visit at my office…

1. … are able to cope with the treatment and compliance.

   1☐ ------- 2☐ ------- 3☐ ------- 4☐ ------- 5☐ ------- 6☐ ------- 7☐

   definitely no *definitely yes*

2. … think they can influence how they'll feel in the future.

   1☐ ------- 2☐ ------- 3☐ ------- 4☐ ------- 5☐ ------- 6☐ ------- 7☐

   definitely no *definitely yes*

3. … understand that their active participation in treatment and disease prevention is important for their functioning and performance.

   1☐ ------- 2☐ ------- 3☐ ------- 4☐ ------- 5☐ ------- 6☐ ------- 7☐

   definitely no *definitely yes*

Translation only

© PRACTA Team, WUM/NTNU
