# Supplementary figures and images for: Enhancing Doctors’ Competencies in Communication With and Activation of Older Patients: The Promoting Active Aging (PRACTA) Computer-Based Intervention Study
Source: J Med Internet Res. 2017 Feb 22;19(2):e45. doi: 10.2196/jmir.6948 (PMC5343213; doi:10.2196/jmir.6948)

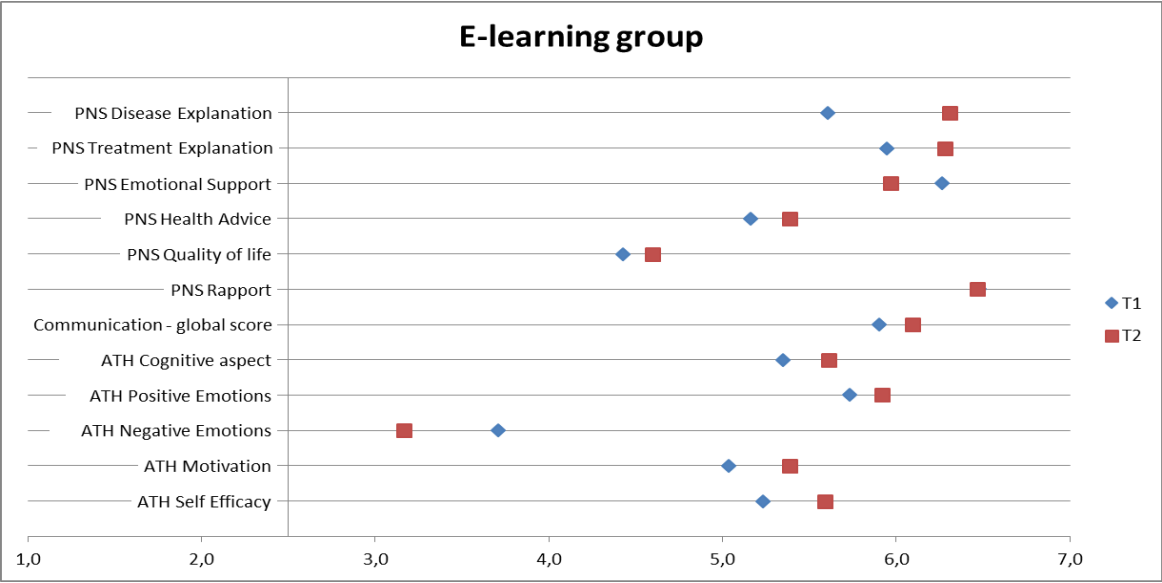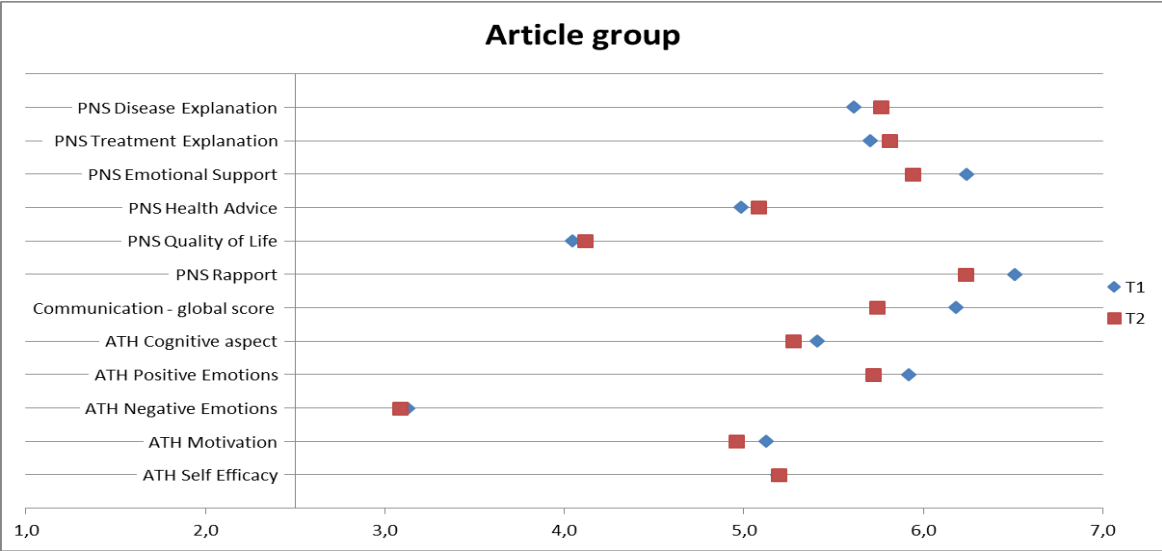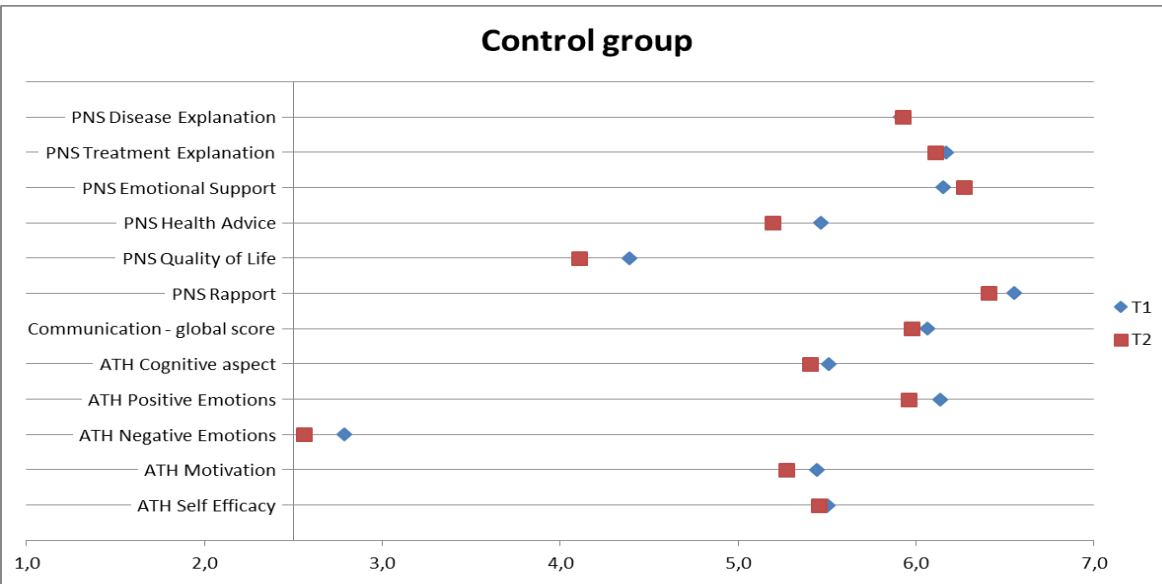

Supplement: Multimedia Appendix 5 [file jmir_v19i2e45_app5.pdf]
